# Supplementary material for: Skyrmion phase and competing magnetic orders on a breathing kagomé lattice
Source: Nat Commun. 2019 Dec 24;10:5831. doi: 10.1038/s41467-019-13675-4 (PMC6930224; doi:10.1038/s41467-019-13675-4)
Supplement: Supplementary file 1 — Supplementary Information [file 41467_2019_13675_MOESM1_ESM.pdf]

Supplementary Information: Skyrmion phase and competing magnetic orders on a breathing kagomé lattice.

Max Hirschberger[1,*] Taro Nakajima[1,†] Shang Gao[1], Licong Peng[1], Akiko Kikkawa[1], Takashi Kurumaji[1,‡] Markus Kriener[1], Yuichi Yamasaki[2,3], Hajime Sagayama[4], Hironori Nakao[4], Kazuki Ohishi[5], Kazuhisa Kakurai[1,5], Yasujiro Taguchi[1], Xiuzhen Yu[1], Taka-hisa Arima[1,6], Yoshinori Tokura[1,7]

[1]*RIKEN Center for Emergent Matter Science (CEMS),Wako 351-0198, Japan*

[2]*Research and Services Division of Materials Data and Integrated System (MaDIS), National Institute for Materials Science (NIMS), Tsukuba 305-0047, Japan*

[3]*PRESTO, Japan Science and Technology Agency (JST), Kawaguchi 332-0012, Japan*

[4]*Institute of Materials Structure Science, High Energy Accelerator Research Organization, Tsukuba, Ibaraki 305-0801, Japan*

[5]*Neutron Science and Technology Center, Comprehensive Research Organization for Science and Society (CROSS), Tokai, Ibaraki 319-1106, Japan*

[6]*Department of Advanced Materials Science, University of Tokyo, Kashiwa, Chiba 277-8561, Japan*

[7]*Department of Applied Physics, University of Tokyo, Bunkyo-ku 113-8656, Japan*

(Dated: October 1, 2019)

*Electronic address: maximilian.hirschberger@riken.jp

†Electronic address: taro.nakajima@riken.jp

‡Current address: Department of Physics, Massachusetts Institute of Technology, Cambridge, Massachusetts 02139, USA

Supplementary Note 1.

RIETVELD REFINEMENT OF CRYSTAL STRUCTURE

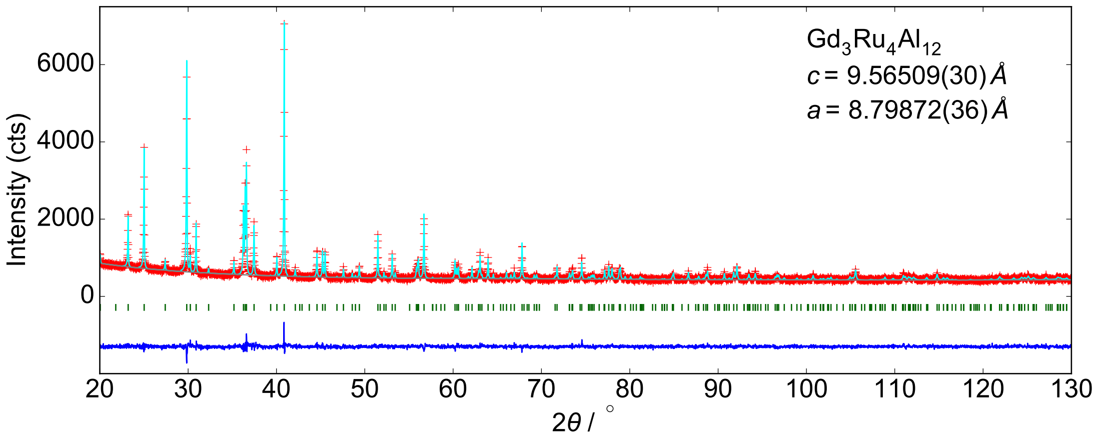

Supplementary Fig. 1: Full refinement of x-ray powder diffraction (XRD) spectrum for $Gd_3Ru_4Al_{12}$. The data were analyzed using the RIETAN software, with fit quality estimated through $R_{wp} = 4.986$, and $S = 1.1643$.

A full refinement of the crystal structure of our $Gd_3Ru_4Al_{12}$ single crystals, crystallizing in space group $P6_3/mmc$ (#194), was performed using x-ray powder diffraction (XRD) data and the software RIETAN [1]. The XRD pattern was taken with a commercial in-house diffractometer (Rigaku SmartLab) at room temperature. An incident monochromatized x-ray beam with wavelength of 1.540593 Å was obtained by Cu $K_{\alpha 1}$ radiation.

Our refined lattice parameters are in rough agreement with the results of previous work [2][3][4][5]. Small deviations in crystal stoichiometry may affect the exact values of the lattice constants, leading to variations of the refined a and c values beyond the error bars provided by these authors. In contrast to a report by Niermann and Jeitschko, who refined a small amount of Ru/Al mixing on the Al site at Wyckoff 6h for $Y_3Ru_{4.060(3)}Al_{11.940(3)}$ [3], our x-ray data for isostructural $Gd_3Ru_4Al_{12}$ is well reproduced by assuming 100 % site occupancy at all positions.

Atom	Wyckoff	coordinates	x	y	z	B
Gd1	6h	$(x, 2x, 1/4)$	0.19287	$2x$	0.25	0.778
Ru1	2a	$(0, 0, 0)$	0	0	0	0.630
Ru2	6g	$(1/2, 0, 0)$	0.5	0	0	0.630
Al1	6h	$(x, y, 1/4)$	0.55845	$2x - 1$	0.25	0.216
Al2	12k	$(2y, y, z)$	$2y$	0.16350	0.42343	0.216
Al3	4f	$(1/3, 2/3, z)$	0.33333	0.66667	0.01550	0.216
Al4	2b	$(0, 0, 1/4)$	0	0	0.25	0.216

Supplementary Table 1: Refined structural parameters of powder x-ray diffraction of stoichiometric $Gd_3Ru_4Al_{12}$. The corresponding fit pattern is shown in Supplementary Fig. 1. B is the anisotropic displacement parameter, while x, y, and z are coordinates of atoms in the unit cell as fractions of the lattice parameters a, b, and c, respectively. Some atomic coordinates are fixed by symmetry (c.f. coordinates column).

Supplementary Note 2.

EVIDENCE FOR MULTI-DOMAIN GROUND STATE

In addition to the SANS data presented in the Methods section, we further used the high-angle detector bank at TAIKAN to investigate the effect of in-plane magnetic field $\mathbf{H} \parallel \mathbf{a}^*$ on the neutron scattering intensities. The color maps of Supplementary Fig. 2 (a-g) show constant-Q_z cuts of the neutron scattering data, which approximately correspond to intensity distributions in the $(H, K, 2)$ plane. Note that the origin of the Q_x-Q_y plane is shifted to the half-way point between the two $(0, 0, 2)$ reflections attributed to different crystallographic domains. In this configuration, energetic considerations lead us to expect that the helical domain with $\mathbf{q} \parallel \mathbf{H}$ will grow in volume as the field is applied. We follow the incommensurate scattering intensity at three magnetic reflections starting from the zero-field cooled state at $T = 2.4\,\mathrm{K}$. The relevant reflections are marked by white circles in Supplementary Fig. 2 (a-g). The integrated scattering intensity at these reflections, shown in Supplementary Fig. 2 (i), exhibits the expected enhancement of intensity for $\mathbf{q} \parallel \mathbf{H}$, as well as finite hysteresis of the intensities after cycling back to zero field. This hysteretic behavior indicates that the zero-field ground state is multi-domain. We note that again, additional reflections appear in

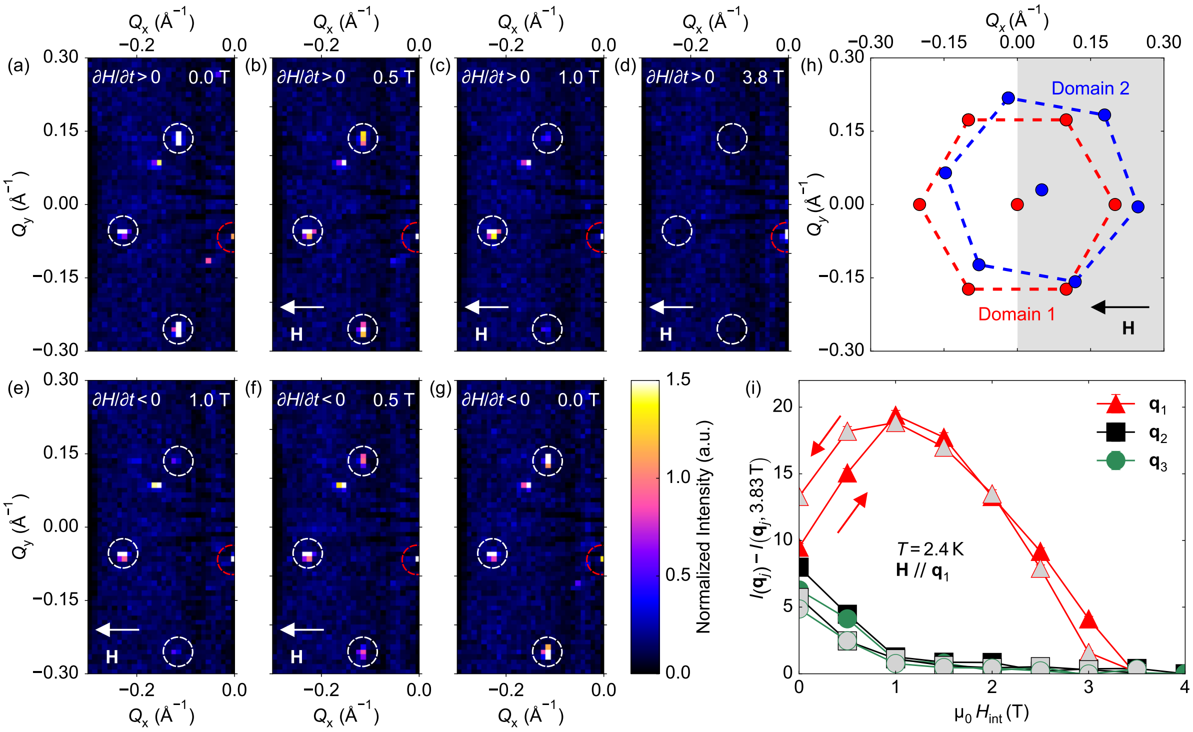

Supplementary Fig. 2: (color online). Evidence for multi-domain nature of helical order in zero magnetic field and $T = 2.4\,\mathrm{K}$. (a-d), Starting from the zero-field cooled state, we apply a magnetic field in the hexagonal basal plane and stabilize a single-domain incommensurate state. Magnetic field direction is shown by a white arrow. Reflections at $\mathbf{q}_1$, $\mathbf{q}_2$, and $\mathbf{q}_3$, are marked by white circles, and the $(0, 0, 2)$ Bragg peak is circled in red. (e-g), Upon releasing the magnetic field amplitude, the intensity returns at all three $\mathbf{q}_i$. The result is summarized in (i), showing hysteresis of the scattering amplitudes evidencing a multi-domain state. (h), Neutron scattering intensity from two crystallographic domains was observed in our ^{160}Gd enriched sample. We focus on domain 1, i.e. the reflections marked by white circles in (a-g). See text for definition of $\mathbf{Q}$ and its origin in this data set. Demagnetization correction has been applied to the field values shown in this figure.

the detector map due to the crystallographic minority domain in our sample (Supplementary Fig. 2 (h)). The misalignment of the crystallographic domains is more apparent in this data set, taken for $\mathbf{q}$-reflections around the $(0, 0, 2)$ zone center, as compared to the SANS data of Fig. 5.

Supplementary Note 3.

FERROMAGNETIC COUPLING BETWEEN BREATHING KAGOMÉ LAYERS

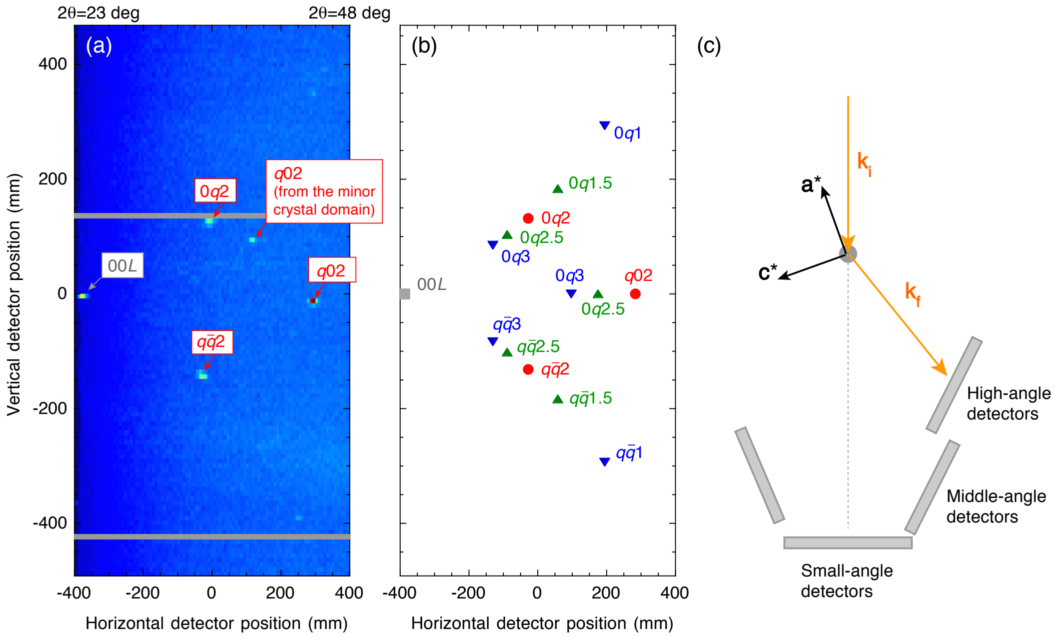

Supplementary Fig. 3: (color online). Absence of antiferromagnetic reflections in zero-field elastic neutron scattering ($T = 2.4\,\mathrm{K}$). (a), High angle detector map shows reflections from two domains, which are labeled with their (h, k, l) indices in the figure. (b), Comparison with a calculation of expected reflections for ferromagnetic coupling of all layers stacked along the c-axis (red circles, observed), antiferromagnetic coupling between adjacent unit cells (green triangles, not observed), and antiferromagnetic coupling between bilayers in a single unit cell (blue triangles, not observed). (c), Sketch of scattering geometry as used in this experiment, exploiting the high-angle detector bank at TAIKAN for the in-plane magnetic field configuration. $\mathbf{k}_i$ and $\mathbf{k}_f$ are the wave vectors of incoming and outgoing beams, respectively. $\mathbf{a}^*$ and $\mathbf{b}^*$ label directions in reciprocal space.

Supplementary Fig. 3 (a,b) shows observed and calculated neutron Laue diffraction patterns at $T = 2.4\,\mathrm{K}$ in zero magnetic field using the high-angle detector bank of TAIKAN (Supplementary Fig. 3 (c)). Magnetic reflections were absent at the $(q, 0, (2n-1)/2)$ and $(q, 0, 2n-1)$ positions ($n = 1, 2$), suggesting that the magnetic modulations in neighboring kagomé layers are coupled in-phase. This analysis confirms that in $Gd_3Ru_4Al_{12}$, antifer-

romagnetic coupling between bilayers is not present in the ground state. Note that in a recent neutron scattering experiment for the sister compound $Dy_3Ru_4Al_{12}$, commensurate $\mathbf{q} = (1/2, 0, 1/2)$ order was refined in the zero field ground state, indicating a doubling of the magnetic unit cell along the c-axis [6].

Supplementary Note 4.

EFFECTIVE EXCHANGE INTERACTIONS IN $GD_3RU_4AL_{12}$

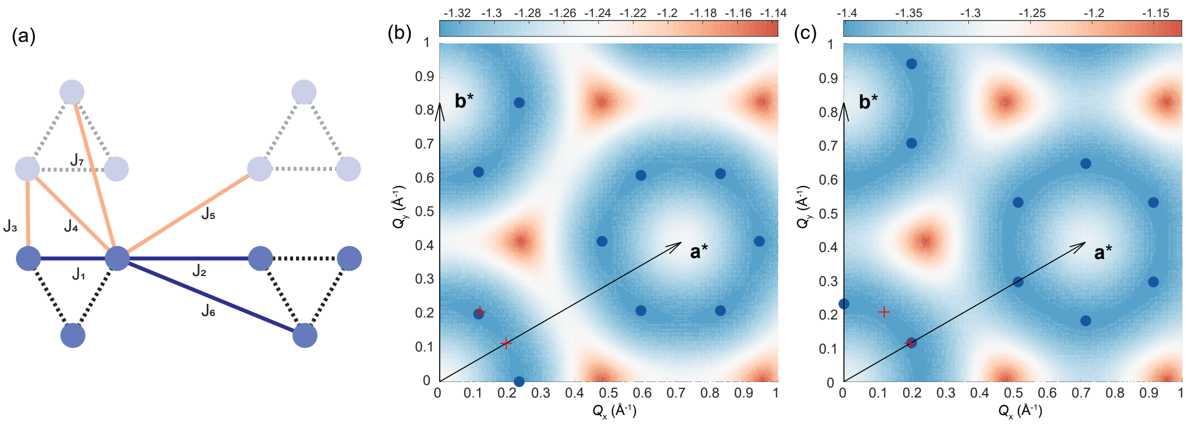

Supplementary Fig. 4: Effective spin model reproducing the magnetic ordering vector in $Gd_3Ru_4Al_{12}$. (a) Magnetic couplings in the breathing kagomé lattice of $Gd_3Ru_4Al_{12}$ up to the seventh nearest-neighbor. Gd ions in upper and lower layers are shown in dark and light blue color, respectively. Intra-layer (inter-layer) couplings are shown as blue (red) lines. (b,c) Calculated minimal eigenenergy map in the $(h, k, 0)$ plane with (b) $J_1 = -1$, $J_2 = 0.25$, $J_7 = 0.125$ and (c) $J_1 = -1$, $J_2 = 0.25$, $J_3 = 0.05$, $J_5 = -0.05$, $J_7 = 0.125$. Positions within the Brillouin zone with the lowest eigenenergy are marked by blue dots. The red crosses indicate the positions of $\mathbf{q}_1 = (0.275, 0, 0)$ and $\mathbf{q}_2 = (0.167, 0.167, 0)$.

In the case of $Gd_3Ru_4Al_{12}$, we start from an effective spin model and search for the ground state configuration under the mean-field approximation. Such a Hamiltonian has the well-known general form (assuming Heisenberg moments)

$$\mathcal{H} = \sum_{i,j} J_{ij}\, \mathbf{S}_i \cdot \mathbf{S}_j \tag{1}$$

where $\mathbf{S}_i$ are local moment spins, J_{ij} are coupling constants, and the sum runs over all pairs of local moments at lattice sites i, j. In our quest for a minimal effective model reproducing the

experimentally observed **q**-vector in this structure, we found that nearest neighbor $J_1 = -1$ (ferromagnetic coupling on Gd triangles, $T_{CW} > 0$), $J_2 = 0.25$, and $J_7 = 0.125$ yield the correct magnitude $q = 0.275$ r.l.u., but aligned along the a-axis as $\mathbf{q} = (0.167, 0.167, 0)$ (Supplementary Fig. 4 (a)). Here, the absolute magnitude of J_2 was chosen assuming exponential decay of the coupling strength in real space, i.e. $|J_2/J_1| = \exp(-d_2/d_1)$. The distances d_1 and d_2 are nearest neighbor and next-nearest neighbor spacings between magnetic sites in the crystal structure of $Gd_3Ru_4Al_{12}$. We stress that the contour plot of eigenenergies in the $q_z = 0$ plane shows a valley (a ring) of nearly degenerate **q** of equal length. However, following the inclusion of two additional small terms $J_3 = 0.05$ and $J_5 = -0.05$, the scattering data for $Gd_3Ru_4Al_{12}$ can be reproduced viz. $\mathbf{q} = (0.275, 0, 0)$ (Supplementary Fig. 4 (c)).

The simpler choice of $J_1 = -1$ paired with any $J_2 > 0$ and *no higher order terms* yields $\mathbf{q} = (1/3, 1/3, 0)$, the characteristic ordering vector of a triangular lattice of antiferromagnetically coupled magnetic trimers. This is in obvious disagreement with the experimental observation.

The crucial point here is not the exact choice of exchange parameters, but the fact that a minimal model well reproduces the near-degeneracy of different directions of **q**. This may provide a framework in which to understand the seemingly disparate results for the direction of the **q**-vector in thin plate and bulk experiments, when taking into account small perturbations such as local lattice strain, surface pinning and impurities, and the effects of the demagnetization field in the thin plate. In support of our conclusion, we note that our most recent scattering experiments on slightly doped $Gd_3(Ru_{1-x}Rh_x)_4Al_{12}$ have shown that $\mathbf{q} = (0.275, 0, 0)$ at $x = 0$ is rotated to $\mathbf{q} = (0.167, 0.167, 0)$ at $x = 0.03 - 0.04$, where T_{N2} and T_{CW} endure only moderate changes [7]. Finally, we note that similar rotations of the magnetic ordering vector by 30° are commonplace in non-centrosymmetric compounds hosting skyrmions, such as the chiral magnetic insulator Cu_2OSeO_3 [8] and the $Co_8Zn_8Mn_4$ alloy [9].

Supplementary Note 5.
ADDITIONAL DATA FOR RESISTIVITY AND HALL EFFECT

Resistivity ρ_{xx} vs. temperature T curves show clear kinks at the two magnetic ordering transitions T_{N1} and T_{N2} (Supplementary Fig. 5 (b)), when fluctuations begin to freeze

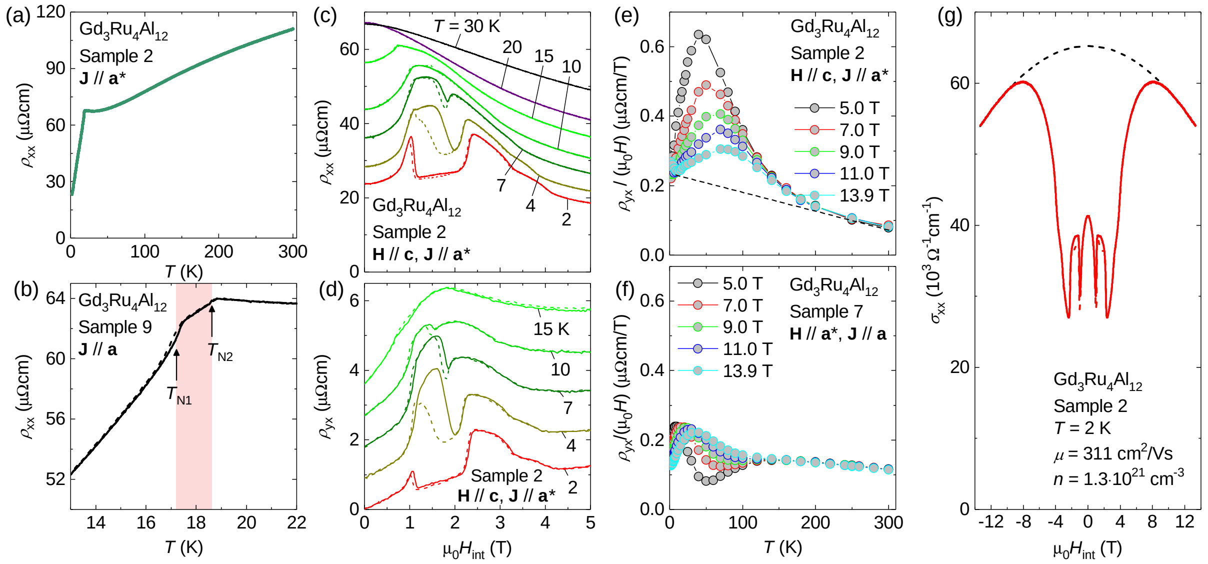

Supplementary Fig. 5: Supporting transport experiments on $Gd_3Ru_4Al_{12}$. (a), Resistivity ρ_{xx} as a function of T for sample 2 (discussed in the main text), in zero magnetic field. (b), For the bar shaped sample 9, high resolution transport data was obtained around the magnetic transition temperatures T_{N1} and T_{N2}. Red shaded area marks the regime of sinusoidal magnetic order (bounded by T_{N1}, T_{N2}). (c,d), For sample 2, raw data of $\rho_{xx}(H)$ and Hall resistivity $\rho_{yx}(H)$ show strong changes due to variations of the scattering time τ. Such data were used to calculate the Hall conductivity shown in Fig. 2. (e), Reduced value $\rho_{yx}/(\mu_0 H)$ of the Hall resistivity for $\mathbf{H} \parallel \mathbf{c}$. Strong anomalous Hall effect (AHE) below $T = 150\,\mathrm{K}$ leads to deviation from the linear high-T behavior. The normal Hall effect is estimated by the black dashed line. (f), Identical analysis for another sample, which was cut so that $\mathbf{H} \parallel \mathbf{a}^*$ could be applied. The temperature dependence of the Hall effect is much weaker in this geometry, due to smaller value of the AHE for in-plane magnetic field. (g), Longitudinal electrical conductivity σ_{xx} is quadratic at high magnetic field, where the magnetic moments are roughly co-aligned. Dashed line is a quadratic fit to the data above $\mu_0 H = 11\,\mathrm{T}$, from which estimates of carrier mobility μ and carrier density n were obtained.

out at the onset of long-range magnetic order. We observed weak hysteresis below T_{N1} between $\rho_{xx}(T)$ recorded with increasing and decreasing temperature, suggesting that this boundary between sinusoidal and helical states is weakly first order. This is consistent with weak hysteresis in the $M - T$ curves (Fig. 1 (e)). We could not resolve hysteresis in the measured observables at T_{N2}. In Supplementary Fig. 5 (c,d), we show raw data of $\rho_{xx}(H_{\mathrm{int}})$

and the Hall resistivity $\rho_{yx}(H_{\text{int}})$, from which the Hall conductivity traces $\sigma_{xy}(H_{\text{int}})$ of Fig. 2 (b) were calculated according to $\sigma_{xy} = \rho_{yx}/(\rho_{xx}^2 + \rho_{yx}^2)$. Strong changes of the carrier lifetime, related to a series of magnetic transitions, affect these observables: While ρ_{xx} deep inside the transverse conical state is comparable to the zero-field ground state, it is possible that domain wall scattering and phase mixing around the metamagnetic critical fields cause enhanced elastic scattering. The high value of ρ_{xx} inside the fan state, similar in magnitude and shape to the paramagnetic state, is notable as well. These changes of the carrier mobility are also reflected in the Hall resistivity. For example, in the case of a purely intrinsic mechanism for the anomalous Hall conductivity, we have $\sigma_{xy}^{\text{AHE}} = S_H M$ and $\rho_{yx}^{\text{AHE}} \sim \sigma_{xy}^{\text{AHE}} \cdot \rho_{xx}^2$ (limit of small Hall angle, $\rho_{xx} \gg \rho_{yx}$) [10]. The resulting non-linearities of ρ_{yx} complicate the separation of the topological Hall resistivity ρ_{yx}^{THE} arising from the scalar spin chirality of magnetic skyrmions in the SkL phase. We address this issue by using σ_{xy} to subtract the smooth background and isolate the topological signal, instead of directly analyzing ρ_{yx}.

In order to discuss the normal Hall coefficient R_0, we consider the temperature dependent Hall resistivity for two samples of different geometry in Supplementary Fig. 5. These samples were cut to be thin plates. Consider a Cartesian coordinate frame where the $x - y$ plane is parallel to the face of the plate, and the current density is $\mathbf{J} \parallel \mathbf{x}$. The magnetic field is $\mathbf{H} \parallel \mathbf{z}$. Sample 2 was cut with $\mathbf{x} = \mathbf{a}^*$ and $\mathbf{y} = \mathbf{b}$ ($\mathbf{H} \parallel \mathbf{c}$), while we prepared sample 7 with $\mathbf{x} = \mathbf{a}$ and $\mathbf{y} = \mathbf{c}$ ($\mathbf{H} \parallel -\mathbf{b}^*$). Supplementary Fig. 5 (e,f) shows $\rho_{yx}(T)/(\mu_0 H)$, a quantity which emphasizes the onset of non-linear Hall resistivity by a divergence of curves measured at different values of the magnetic field. This point of departure is found at lower T in sample 7 ($\mathbf{H} \parallel -\mathbf{b}^*$), where the anomalous Hall effect (AHE) is suppressed as compared to the case of $\mathbf{H} \parallel \mathbf{c}$. We infer that R_0, which should be comparable for the two sample geometries in the case of a roughly three-dimensional Fermi surface, and which is expected to dominate at $T = 300\,\text{K}$, is only weakly dependent on T. In the case of $\mathbf{H} \parallel \mathbf{c}$, the strong temperature dependence of ρ_{yx} at $\mu_0 H_{\text{int}} > 3\,\text{T}$ thus arises mostly due to the AHE from spin-orbit coupling, and possibly from scalar spin chirality on isolated Gd plaquettes [2]. As expected in the framework of the intrinsic mechanism, which has ρ_{yx}^{AHE} strongly dependent on the scattering time τ [10], the AHE dies off at the lowest temperatures. The nature of the high-field AHE in $R_3\text{Ru}_4\text{Al}_{12}$ (R: rare earth) and its anisotropy will be the subject of a future study. Exploiting the weak temperature dependence of R_0, we use the extrapolated

Hall coefficient $R_0 = 0.2\,\mu\Omega\mathrm{cm/T}$ (dashed line in Supplementary Fig. 5 (e)) in our analysis of the emergent magnetic field B_{em} of the SkL (c.f. main text).

What follows is an order-of-magnitude estimation of charge carrier densities and carrier mobilities in $Gd_3Ru_4Al_{12}$. Starting from the semiclassical Drude model [11], we assume very crudely that the various bands may be described by an effective mobility $\bar{\mu}$, so that (electron charge e, magnetic field $B = \mu_0 H$, carrier densities n_j, where the index j runs over all partially occupied bands) $\sigma_{xx} = e\bar{\mu}\sum_j |n_j|/(1+(\bar{\mu}B)^2)$ and $\sigma_{xy} = e\bar{\mu}^2 B\sum_j n_j/(1+(\bar{\mu}B)^2)$. Expanding these expressions to first order in $\bar{\mu}B \ll 1$ and using a quadratic fit to $\sigma_{xx}(H)$ at $\mu_0 H > 11\,\mathrm{T}, T = 2\,\mathrm{K}$ we obtain $\bar{\mu} = 311\,\mathrm{cm}^2/(\mathrm{Vs})$ and $\sum_j |n_j| = 1.3{\cdot}10^{21}\,\mathrm{cm}^{-3}$. Let us cross-check using the Hall resistivity: We have $\rho_{yx} = \sigma_{xy}/(\sigma_{xx}^2+\sigma_{xy}^2) \sim B\sum_j n_j/(e(\sum_j |n_j|)^2)$ for $\sigma_{xx}^2 \gg \sigma_{xy}^2$ and experimentally $\rho_{yx}/(\mu_0 H) \sim 0.20\,\mu\Omega\mathrm{cm/T}$ at $T = 2\,\mathrm{K}$ and high magnetic field (Supplementary Fig. 5 (e)). The demagnetization correction is not included in these back-of-the-envelope estimates. We arrive at $\sum_j n_j = 5.5{\cdot}10^{20}\,\mathrm{cm}^{-3}$; provided the approximations made in the derivation, we cannot draw a conclusion about the number and character of bands contributing to transport. However, weak temperature dependence of R_0 is in principle consistent with conduction from a single carrier type, as is the relatively low absolute value of the carrier density (c.f. $8.5{\cdot}10^{22}\,\mathrm{cm}^{-3}$ for Cu). Under the assumption of a single band with spherical Fermi surface, the Fermi wave vector is estimated as $k_F = (6\pi^2 n/d_s)^{1/3} = 3.4\,\mathrm{nm}^{-1}$, in agreement with the analysis of Ref. [2]. Here, $d_s = 2$ is the spin degeneracy of the band, and n is the total carrier density.

Supplementary Note 6.

REPRODUCIBILITY OF TRANSPORT DATA - HALL EFFECT IN THE SKL PHASE

We have reproduced the topological Hall effect and transport data for a second sample of $Gd_3Ru_4Al_{12}$ from the same growth, with slightly different sample geometry ($\mathbf{J} \parallel \mathbf{a}$, $\mathbf{H} \parallel \mathbf{c}$ for sample 8 instead of $\mathbf{J} \parallel \mathbf{a}^*$, $\mathbf{H} \parallel \mathbf{c}$ for sample 2). The results are in good qualitative agreement (Supplementary Fig. 6), although the boundary between the TC and SkL phases is located at slightly lower temperature in sample 8. Pinning from defects may affect the exact character of the first-order boundary between these two field-induced phases.

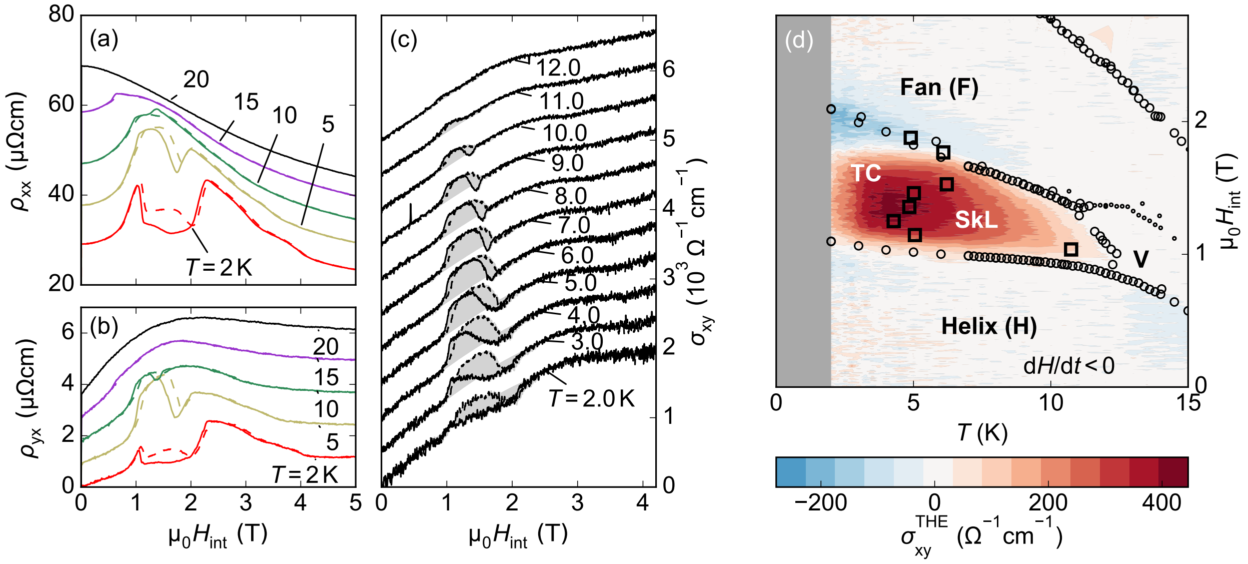

Supplementary Fig. 6: Transport data and topological Hall effect in $Gd_3Ru_4Al_{12}$ sample 8. (a), Magnetoresistance $\rho_{xx}(H)$ with $\mathbf{H} \parallel \mathbf{c}$ and $\mathbf{J} \parallel \mathbf{a}$ has anomalies associated with phase transitions below $T = 20\,\mathrm{K}$. (b), Hall resistivity ρ_{yx} mimics the behavior of ρ_{xx}, both observables being related to the effective carrier mobility μ. (c), Hall conductivity σ_{xy} exhibits smooth background due to normal and anomalous Hall effect. Superimposed, we find a box-shaped, hysteretic topological Hall signal. (d), Topological Hall conductivity $\sigma_{xy}^{\mathrm{THE}}$ is constrained to the SkL phase. Black open circles in (d) are phase boundaries obtained in susceptibility measurements (c.f. Fig. 2 (a)). Transport results are generally comparable to those of sample 2 presented in the main text.

Supplementary Note 7.

MAGNETIZATION ISOTHERMS AND MAGNETIC ANISOTROPY IN $\mathbf{GD_3RU_4AL_{12}}$

In our single crystals, the magnetization vs. magnetic field $(M-H)$ isotherms at $T = 2\,\mathrm{K}$ (Supplementary Fig. 7 (a)) are anisotropic in the low-field regime, with higher magnetization observed along the in-plane (a^*) direction as compared to magnetic field $\mathbf{H} \parallel \mathbf{c}$. This is despite the quenched orbital moment of the Gd $4f^7$ magnetic shell. The saturation value of M, roughly independent of the field direction, significantly exceeds the value expected for free Gd^{3+} ions ($7\mu_B$/Gd, where μ_B is the Bohr magneton). This may be interpreted as a finite orbital component on the Gd site or, alternatively, as a magnetic moment of up to

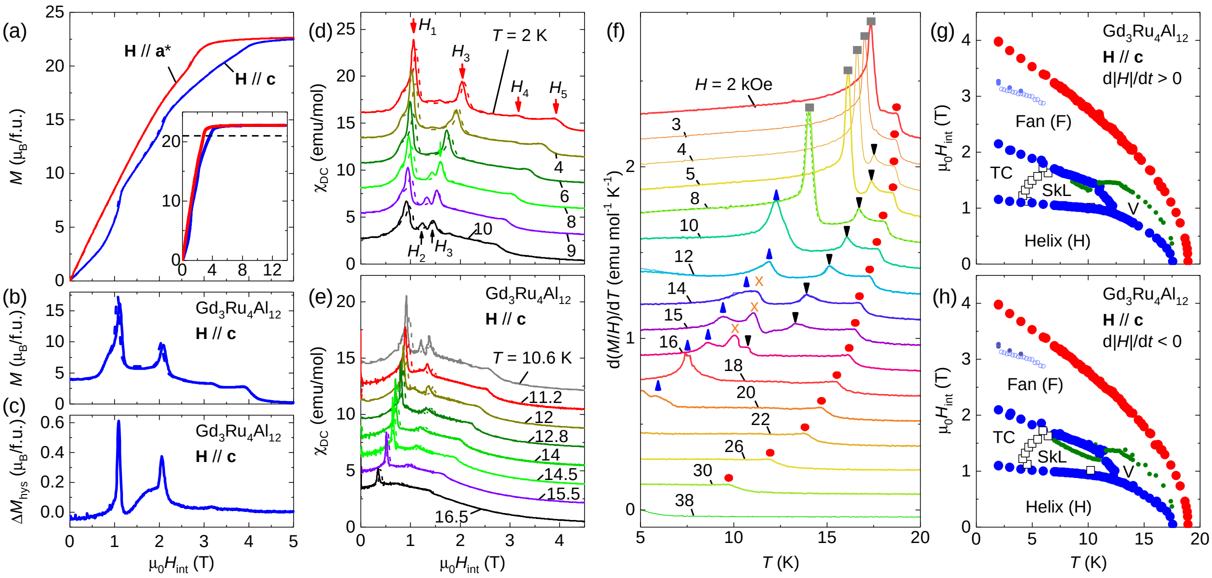

Supplementary Fig. 7: Supplementary magnetization data for $Gd_3Ru_4Al_{12}$. (a), Magnetization isotherms, measured in a vibrating sample magnetometer (VSM), for two directions of magnetic field **H** at $T = 2\,K$. Inset: high-H part (zoom-out). Dashed line indicates expected saturation value of Gd^{3+} free moment. (b,c), DC susceptibility calculated as $\chi_{DC} = \partial M/\partial H$, and hysteretic component of the magnetization $\Delta M_{hyst} = M_{down} - M_{up}$ for $\mathbf{H} \parallel \mathbf{c}$, at $T = 2\,K$. (d,e), Raw data of magnetic susceptibility, used to obtain the contour plot shown in Fig. 2 (a). For $T = 2\,K$ in (d), several signatures associated with phase boundaries in Fig. 2 (a) are highlighted by red arrows. See text for definition of critical fields H_1 - H_5. Dashed lines correspond to increasing, solid lines to decreasing magnetic field. Data offset by constant values for clarity. (f), Temperature derivative of magnetization data $M(T)$ at fixed H with $\mathbf{H} \parallel \mathbf{c}$. Data recorded with decreasing temperature ($\mathrm{d}T/\mathrm{d}t < 0$). Starting at high T: red circles - transition from paramagnetic to fan state; black triangles - fan state to phase V; grey squares - transition to the helical state; blue (up) triangles - transition to SkL phase; orange X marker - possible additional phase boundary which remains unconfirmed. (g,h), Phase diagrams obtained from magnetization measurements, for $\mathrm{d}H/\mathrm{d}t > 0$ and $\mathrm{d}H/\mathrm{d}t < 0$, respectively. Black open square symbols indicate the departure of FC and ZFC curves in the T-dependence of σ_{xy} (c.f. Fig. 2). Light blue solid symbols correspond to H_4 from $M(H)$, while light blue open symbols were obtained from $\rho_{xx}(H)$. Demagnetization correction was applied to all panels with the exception of (f). Curves in (f) were offset by constants for visibility.

$0.4\,\mu_B$/Ru on the ruthenium site (between the kagomé layers). Note that the fluctuating moment $\mu_{\rm eff}$, as obtained from the Curie-Weiss law, also shows some excess over the expected $7.85\,\mu_B$/Gd.

In the case of $\mathbf{H} \parallel \mathbf{c}$, the hysteretic nature of the phase boundaries from the zero-field helical state to transverse conical and further to the fan-like state points towards strong first-order transitions, while the transition from the incommensurate state to the fully field-aligned state appears to be second order (Supplementary Fig. 7 (c)). For the SkL phase, we can conclude that it is bounded by first order phase transitions on all sides. In addition to the magnetic phase boundaries discussed in the main text, we here mention two further anomalies: First, a weaker peak in χ_{DC} is present at elevated temperatures (e.g. $T = 10\,$K in Supplementary Fig. 7 (d)) within the boundaries of the SkL phase. This signature is labeled as H_2 in Supplementary Fig. 7 (d) and indicated by solid, small green symbols in the phase diagrams of Supplementary Fig. 7 (g,h). This may be related to a possible, yet at the time of writing unconfirmed, subdivision of the SkL phase. Furthermore, a shoulder emerges in $\chi_{DC}(H_{\rm int})$ for $T = 2\,$K, $\mathbf{H} \parallel \mathbf{c}$ (Supplementary Fig. 7 (d), third red arrow from the right hand side). Magnetoresistance data shows a kink at the same value of the internal magnetic field H_4, and we can track this critical field up to about $T = 7\,$K, where it grows very weak. We have marked H_4 with small, solid blue circles in Supplementary Fig. 7 (g,h). As we were unable to determine H_4 at high temperature, it remains unclear whether this field scale corresponds to a thermodynamic phase boundary or merely to a cross-over field.

Finally, we note that the signatures that characterize the transition between the fan state and phase V are rather weak as compared to other phase transitions. This transition is most readily observed in the $M-T$ curves, while it leaves no obvious trace in transport experiments. We have also found that signatures associated with phase V are sensitive to disorder. For example, our ^{160}Gd enriched sample with enhanced impurity concentration did not show any anomalies for phase V, while phases H, TC, SkL, and F were clearly identified. Similarly, phase V was not observed in thin-plate samples used for TEM measurements. Note that the thin-plate preparation process involves mechanical thinning.

Sample	measurement	shape	m_s	orientation	N	comments
1	magnetization	cube	5.06		0.33	
2	transport	plate		$\mathbf{J} \parallel \mathbf{a}^*$, $\mathbf{H} \parallel \mathbf{c}$	0.87	c.f. Fig. 2
3	heat capacity	cuboid	7.65		0.50	
4	resonant x-ray	thick plate			0.22	
5	TEM thin	plate		$\mathbf{H} \parallel \mathbf{c}$	0.95	
6	neutron scattering	thick plate	16.86		0.62	^{160}Gd enriched
7	transport			$\mathbf{J} \parallel \mathbf{a}$, $\mathbf{H} \parallel -\mathbf{b}^*$		
8	transport			$\mathbf{J} \parallel \mathbf{a}$, $\mathbf{H} \parallel \mathbf{c}$	0.75	
9	transport	bar		$\mathbf{J} \parallel \mathbf{a}$		

Supplementary Table 2: Single crystalline samples used in this study. Symbols used here are: m_s for sample mass in milligrams, **J** for current density, **H** for internal magnetic field, and N for the demagnetization factor. In all cases, N is given for $\mathbf{H} \parallel \mathbf{c}$. For some samples, the demagnetization factor was not determined. **a**, **c**, and $\mathbf{a}^*$ are crystallographic directions in real and reciprocal space.

SUPPLEMENTARY REFERENCES

[1] Izumi, F. & Momma, K., *Three-Dimensional Visualization in Powder Diffraction.* Solid State Phenomena, **130**, 15-20 (2007)

[2] S. Nakamura, S., Kabeya, N., Kobayashi, M., Araki, K., Katoh, K. & Ochiai, A. *Spin trimer formation in the metallic compound $Gd_3Ru_4Al_{12}$ with a distorted kagome lattice structure.* Physical Review B **98**, 054410 (2018)

[3] Niermann, J. & Jeitschko, W. *Ternary Rare Earth (R) Transition Metal Aluminides $R_3T_4Al_{12}$ (T = Ru and Os) with $Gd_3Ru_4Al_{12}$ Type Structure.* Journal of Inorganic and General Chemistry, **628**, 2549-2556 (2002)

[4] Gladyshevskii, R.E., Strusievicz, O.R., Cenzual, K. & Parthé, E., *Structure of $Gd_3Ru_4Al_{12}$, a new member of the $EuMg_{5.2}$ structure family with minority-atom clusters.* Acta Crystallographica Section B **49**, 474-478 (1993)

[5] Chandragiri, V., Iyer, K. K. & Sampathkumaran, E.V. *Magnetic behavior of $Gd_3Ru_4Al_{12}$, a*

layered compound with distorted kagomé net. Journal of Physics: Condensed Matter **28**, 286002 (2016)

[6] Gorbunov, D. I., Henriques, M. S., Andreev, A. V., Gukasov, A., Petříček, V., Baranov, N. V., Skourski, Y., Eigner, V., Paukov, M., Prokleška, J. & Gonçalves, A. P. *Electronic properties of a distorted kagome lattice antiferromagnet* $Dy_3Ru_4Al_{12}$. Physical Review B **90**, 094405 (2014)

[7] Hirschberger, M., Nakajima, T., Gao, S., *et al.*, manuscript in preparation

[8] Seki, S., Kim, J.-H., Inosov, D. S., Georgii, R., Keimer, B., Ishiwata, S. & Tokura, Y. *Formation and rotation of skyrmion crystal in the chiral-lattice insulator* Cu_2OSeO_3. Phys. Rev. B **85**, 220406(R) (2012)

[9] Karube, K., White, J. S., Reynolds, N., Gavilano, J. L., Oike, H., Kikkawa, A., Kagawa, F., Tokunaga, Y., Rønnow, H. M., Tokura, Y. & Taguchi, Y. *Robust metastable skyrmions and their triangular-square lattice structural transition in a high-temperature chiral magnet.* Nature Materials **15**, 1237-1242 (2016)

[10] Nagaosa, N., Sinova, J., Onoda, S., MacDonald, A.H., & Ong, N.P. *Anomalous Hall Effect.* Reviews of Modern Physics **82**, 1539 (2010)

[11] Ziman, J.M. *Principles of the Theory of Solids.* 2nd Edition, Cambridge University Press (1972)
